# Development of Self-Nanoemulsifying Drug Delivery Systems Containing 4-Allylpyrocatechol for Treatment of Oral Infections Caused by *Candida albicans*

**DOI:** 10.3390/pharmaceutics13020167

**Published:** 2021-01-27

**Authors:** Siriporn Okonogi, Pimpak Phumat, Sakornrat Khongkhunthian, Pisaisit Chaijareenont, Thomas Rades, Anette Müllertz

**Affiliations:** 1 Department of Pharmaceutical Sciences, Faculty of Pharmacy, Chiang Mai University, Chiang Mai 50200, Thailand; 2 Research Center of Pharmaceutical Nanotechnology, Faculty of Pharmacy, Chiang Mai University, Chiang Mai 50200, Thailand; sakornrat.k@cmu.ac.th (S.K.); pisaisit.c@cmu.ac.th (P.C.); 3 Interdisciplinary Program in Nanoscience and Nanotechnology, Faculty of Science, Chiang Mai University, Chiang Mai 50200, Thailand; pimpak_p@cmu.ac.th; 4 Department of Restorative Dentistry and Periodontology, Faculty of Dentistry, Chiang Mai University, Chiang Mai 50200, Thailand; 5 Department of Prosthodontics, Faculty of Dentistry, Chiang Mai University, Chiang Mai 50200, Thailand; 6 Department of Pharmacy, Faculty of Health and Medical Sciences, University of Copenhagen, 2100 Copenhagen, Denmark; thomas.rades@sund.ku.dk (T.R.); anette.mullertz@sund.ku.dk (A.M.)

**Keywords:** SNEDDS, 4-allylpyrocatechol, solubility enhancement, antifungal activity, oral infections, *Candida albicans*

## Abstract

Clinical use of 4-Allylpyrocatechol (APC), a potential antifungal agent from *Piper betle*, is limited because of its low water solubility. The current study explores the development of the self-nanoemulsifying drug delivery system (SNEDDS) containing APC (APC-SNEDDS) to enhance APC solubility. Results demonstrated that excipient type and concentration played an important role in the solubility of APC in the obtained SNEEDS. SNEDDS, comprising 20% Miglyol 812N, 30% Maisine 35-1, 40% Kolliphor RH40, and 10% absolute ethanol, provided the highest loading capacity and significantly increased water solubility of APC. Oil-in-water nanoemulsions (NE) with droplet sizes of less than 40 nm and a narrow size distribution were obtained after dispersing this APC-SNEDDS in water. The droplets had a negative zeta potential between −10 and −20 mV. The release kinetics of APC from APC-SNEDDS followed the Higuchi model. The NE containing 1.6 mg APC/mL had effective activity against *Candida albicans* with dose-dependent killing kinetics and was nontoxic to normal cells. The antifungal potential was similar to that of 1 mg nystatin/mL. These findings suggest that APC-SNEDDS are a useful system to enhance the apparent water solubility of APC and are a promising system for clinical treatment of oral infection caused by *C. albicans*.

## 1. Introduction

*Candida albicans* is a commensal microbe in the oral cavity of 45–65% of healthy individuals, with a higher prevalence found in children and young adults. Oral fungal infections caused by *C. albicans* constitute a severe health problem. Generally, this microorganism can adapt to its host and colonize various sites without causing notable damage. However, in certain environmental conditions, the overgrowth of this microorganism can occur and lead to a diverse array of diseases, ranging from common mucosal infection to invasive systemic infection, resulting in host-mediated damage [1]. The severity of *C. albicans* in the oral cavity is associated with wearing removable dentures because the dentures can decrease the flow of saliva and oxygen to the tissue covered by the dentures. This leads to a high production of a local acidic and anaerobic environment that favors overgrowth of *C. albicans*. It has been reported that the growth of *C. albicans* in that environment increases by 60–100% [2]. *C. albicans* can thus be categorized as a severe opportunistic pathogen in people wearing dentures. The most common form of oral candidiasis is denture-related stomatitis, which is an inflammatory process in an oral cavity. Previous studies reported that candida-associated denture stomatitis can occur in up to 65% of denture wearers [2,3]. A general hygiene process to prevent pathogenic accumulation on the dentures can be performed by soaking the dentures in denture cleansing solutions containing chemical disinfectants such as nystatin, chlorhexidine, and sodium hypochlorite [4]. However, the use of these substances may be harmful to human health and environment [5]. Natural compounds from plants that possess effective antimicrobial activity are, therefore, of increasing interest as alternative agents for denture-cleansing products.

**Figure 1:**
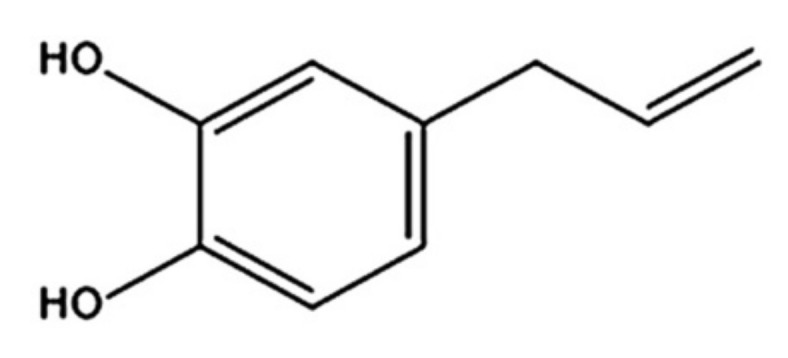
Chemical structure of 4-Allylpyrocatechol (APC).

4-Allylpyrocatechol (APC), chemical structure as shown in Figure 1, is a major compound of *Piper betle*, a member of the Piperaceae family, that possesses several pharmacological activities, including anti-inflammatory and antimicrobial activities [6]. It has a strong antifungal activity against various *Candida* species, including *C. albicans* [7]. In a previous study, we isolated APC from the chloroform extract of *P. betle* leaves and determined its antifungal activity against *C. albicans*. The results confirmed that APC can inhibit *C. albicans* effectively [8]. APC is soluble in moderately nonpolar solvents, and therefore can be found in the chloroform or ethyl acetate extracts of *P. betle* [9,10]. However, APC has a low water solubility. Therefore, its clinical use to combat oral fungal infections is limited.

Lipid-based drug delivery systems (LbDDS) can improve the apparent solubility, miscibility, and oral bioavailability of many poorly water-soluble drugs [11,12]. These systems are generally composed of oils, surfactants, and co-surfactants. According to the excipients in the formulations, LbDDS can be characterized into four types, shown in Table 1, with unique features, advantages, and disadvantages for each type [13,14]. Self-nanoemulsifying drug delivery systems (SNEDDS) are one of the potential LbDDS to promote apparent water solubility of hydrophobic drugs including natural compounds [15]. The homogenous, single-phase lipid mixture of SNEDDS can spontaneously create oil-in-water (o/w) nanoemulsions (NEs) with an approximate particle size of 200 nm or less after being dispersed in water with only gentle agitation [16]. The advantage of SNEDDS over other lipid carriers, such as nanoemulsions, liposomes, solid lipid nanoparticles, and nanostructured lipid carriers [17], is a significantly lower amount of energy required for dispersion and preparation. Another benefit of SNEDDS is their high physical stability upon storage and kinetic stability after dispersing in an aqueous phase [18].

The present study aims to develop and characterize SNEDDS containing APC from *P. betle* (APC-SNEDDS) to enhance the apparent water solubility of APC. Various anhydrous SNEDDS formulations were developed, and the optimized formulation was selected for loading APC. Phase diagrams were used to select the type and concentration of the excipients needed to form the APC-SNEDDS that yielded the most suitable NE composed of soybean oil or Miglyol 812N, Maisine 35-1, Kolliphor RH40, and absolute ethanol. The antifungal activity of the developed APC-SNEDDS against *C. albicans* was investigated by determination of the killing kinetics and by electron microscopy to investigate the effects of APC-SNEDDS on the destruction of *Candida* cells in comparison with nystatin, a polyene macrolide antifungal drug.

## 2. Materials and Methods

### 2.1. Materials

3-(4,5-Dimethylthiazol-2-yl)-2,5-diphenyltetrazolium bromide (MTT) was purchased from Sigma-Aldrich (St. Louis, MO, USA). Miglyol 812N was purchased from Cremer Oleo GmbH & Co. KG (Hamburg, Germany). Maisine 35-1 (a mixture of long-chain mono-, di-, and triglycerides) was kindly donated by Gattefossé (Saint Priest, France). Kolliphor RH40 (polyoxyl 40 hydrogenated castor oil) was purchased from BASF (Ludwigshafen, Germany). Tween 80 (polyoxyethylene (80) sorbitan monolaurate) and Tween 20 (polyoxyethylene (20) sorbitan monolaurate) were purchased from Fluka (Buchs, Switzerland). Absolute ethanol and methanol HPLC grade were purchased from VWR (Radnor, PA, USA). Sabouraud dextrose broth (SDB) and Sabouraud dextrose agar (SDA) were purchased from Difco (Sparks, MD, USA). Glutaraldehyde was purchased from Merck (Darmstadt, Germany). Nystatin was purchased from T.O. Pharma Co., LTD (Bangkok, Thailand). Dulbecco’s modified eagle medium (DMEM), fetal bovine serum (FBS), and antibiotic-antimycotic solution (AA) containing amphotericin B, penicillin, and streptomycin were purchased from Gibco (Burlington, Canada). Complete DMEM; a mixture composed of DMEM, 10% *v*/*v* FBS and 1% *v*/*v* AA, and glacial acetic acid glacial, was from QRëC (Auckland, New Zealand). Other chemicals and solvents were of analytical grade.

### 2.2. High Performance Liquid Chromatography (HPLC) Analysis

Quantitative analysis of APC was performed on an HPLC system composed of a Dionex ASI—100 automated sample injector, PDA—100 photodiode array detector, and P680 HPLC pump (Thermo Fisher Scientific, Waltham, MA, USA). A Phenomenex Luna reversed phase HPLC column C18 (4.6 mm × 250 mm × 5 mm) (Torrance, CA, USA) was used as stationary phase. The samples were dissolved in absolute ethanol and filtered through a 0.22 μm filter (Whatman, Marlborough, MA, USA) before injection with an injection volume of 10 μL. The mobile phase consisted of 70% *v*/*v* methanol and 30% *v*/*v* water and was isocratically pumped at a flow rate of 1 mL/min for 20 min. The eluted samples were detected using a UV/VIS detector (Thermo Fisher Scientific, Waltham, MA, USA) at a wavelength of 280 nm. The retention time of APC was approximately 5.0 min. The amount of APC was calculated from peak areas using a linear standard curve ranging from 100 to 1000 µg/mL.

### 2.3. Solubility of APC in Excipients and in Water

Solubility determination in various excipients was performed to select suitable excipients for the preparation of SNEDDS. An excess amount of APC was added in 100 mg of each excipient (soybean oil, Miglyol 812N, Maisine 35-1, Kolliphor RH40, Tween 80, Tween 20, and absolute ethanol) in 1.5 mL microcentrifuge tubes, followed by mixing using a vortex mixer and equilibration at room temperature for 24 h. Next, the mixtures were centrifuged at 10,400× *g* (Biofuge 15, Heraeus-Sepatech, Osterode, Germany) for 15 min. A total of 10 µL of the supernatant from each excipient was mixed with 990 µL of absolute ethanol to obtain 100-fold dilution samples. The samples were filtered through a 0.22 µm filter. The concentration of APC in each sample was determined using the HPLC analysis as described above. The solubility of APC in each excipient was calculated using the standard curve and the dilution factor to obtain mg APC/g excipient. Excipients that showed a high APC solubility were selected for further studies. The water solubility of APC was also investigated by adding an excess amount of APC (20 mg) into 1 mL of Milli-Q water (Millipore, Billerica, MA, USA) and the solubility was determined as described above.

### 2.4. Experimental Design and Preparation of SNEDDS Formulations

Preparation of SNEDDS formulations was performed according to a method previously described by Ren et al. [19], with some modifications. The suitable excipients selected from the solubility study were used to design their optimum concentration in the SNEDDS formulations using a D-optimal design (Umetric modeling and design (MODDE) software, version 11.0, Umetric, Sweden). Then, the components of the lipid mixtures, including oils and surfactants, were weighted in Teflon-sealed glass vials, and heated to 50 °C until all components were melted. The lipid mixture was stirred on a magnetic stirrer overnight at room temperature and protected from light. Then, a cosolvent was added to the lipid mixture and the mixture was further stirred on a magnetic stirrer overnight at the same conditions as the lipid mixture.

### 2.5. Investigation of Self-Emulsification Efficiency

To evaluate the self-emulsification efficiency of the prepared SNEDDS formulations, an aliquot of 100 mg SNEDDS was dispersed in a beaker containing 10 mL of Milli-Q water (1% *w*/*v* SNEDDS) at ambient temperature with gentle stirring. The self-emulsification efficiency of the SNEDDS samples was visually evaluated and categorized using a five-grade system [20]:

Grade A: A NE with a clear or bluish appearance was rapidly formed within 1 min.
Grade B: A less clear emulsion with a bluish-white appearance was rapidly formed.
Grade C: A fine milky emulsion was formed within 2 min.
Grade D: A dull, grayish-white emulsion with slightly oily appearance was slowly formed (longer than 2 min).
Grade E: Poor or minimal emulsification with large oil globules was present on the surface.

### 2.6. Measurement of Particle Size, Size Distribution, and Zeta Potential

The particle size, size distribution, and zeta potential of the internal phase droplets of the NEs obtained from diluting SNEDDS in Milli-Q water at a ratio of 1:100 (*w*/*v*) were investigated by means of photon correlation spectroscopy (PCS) at 25 °C using a Zetasizer Nano ZS (Malvern Instruments Ltd., Malvern, Worcestershire, UK). For determination of particle size and size distribution, each NE was transferred into a cuvette and measured on a fixed angle of 173°. The particle size was expressed as an average diameter in nm whereas particle size distribution was expressed as polydispersity index (PdI). For determination of the zeta potential, each NE was transferred into DT51070 folded capillary cells (Malvern, Worcestershire, UK) before subjecting to the PCS. The zeta potential of the samples was automatically calculated based on the Smoluchowski equation [21] using the Zetasizer (Malvern, Worcestershire, UK) software version 7.1. All experiments were performed in triplicate.

### 2.7. Solubility of APC in SNEDDS

The solubility of APC in SNEDDS was determined by adding an excess amount of APC in 500 mg of each anhydrous SNEDDS formulations followed by mixing using a vortex mixer. The mixture was left to equilibrate at room temperature for 24 h. Then, the mixtures were centrifuged at 10,400× *g* for 15 min. A total of 10 µL of the supernatant from each SNEDDS was mixed with 990 µL of absolute ethanol and filtered through 0.22 µm nylon membrane. The solubility of APC in each SNEDDS was determined by HPLC analysis as described above. The obtained solubility results were used to contribute to the prediction contour plot of APC solubility in SNEDDS formulations by using MODDE software base on D-optimal design. The SNEDDS formulation that yielded the highest solubility of APC was categorized as the most suitable formulation that could provide the highest drug-loading capacity and was selected for further study.

### 2.8. Preparation and Characterization of APC-SNEDDS

An exact amount of APC was added to the selected SNEDDS to yield a weight ratio of APC:SNEDDS of 1:7 and the SNEDDS were subsequently mixed using a Vortex mixer to get a homogenous mixture. The obtained mixture was then dispersed in Milli-Q water to provide final APC concentrations of 0.4, 0.8, and 1.6 mg/mL. The dispersed droplets of the obtained NEs were characterized for particle size, size distribution, and zeta potential using PCS, as described above.

### 2.9. Morphology Study

Transmission electron microscopy (TEM) was used to investigate the morphology of the dispersed droplets in the NE. APC-SNEDDS were diluted 10–100 times with Milli-Q water and investigated using a JEM-2200FS TEM (JEOL, Tokyo, Japan) operated at an acceleration voltage at 200 kV. The TEM samples were prepared as follows: 10 µL of the NE was carefully dropped on a discharged 200 mesh copper grid coated with formvar and carbon (FCF-200 mesh Cu, Electron Microscopy Sciences, Hatfield, PA, USA). The excess solution was removed by filter paper and left for air-drying at ambient temperature for 5 min. The sample was subsequently stained with a 1% *w*/*v* phosphotungstic solution. After 2 min, the excess solution was removed using filter paper and the grid was then subjected into the TEM.

### 2.10. In Vitro Release

The release of APC-SNEDDS was determined by a dialysis method modified from the study of Tima et al. [22]. An artificial saliva solution pH 7.4 containing sodium chloride, calcium chloride dihydrate, potassium chloride, sodium sulfide nanohydrate, sodium dihydrogen phosphate monohydrateO, and urea was used as releasing medium. Two mL of NE obtained from the developed APC-SNEDDS was pipetted into a pre-swollen dialysis bag (regenerated cellulose tubular membrane with MWCO of 3500; Cellu Sep T1, Siguin, TX, USA). The dialysis bag was tightly closed and immersed into 30 mL of the release medium with stirring at 100 rpm at 37 °C. Samples (3 mL) of the release medium were drawn periodically at time interval of 15, 30, 60, 120, 240, 360, and 480 min. Fresh medium with the same volume was added into the dissolution medium after each sample withdrawal. The amount of APC was determined by HPLC as described above. The percentage cumulative release of APC at time t (C_t_) was calculated using the following Equation (1):

C_t_ = (C_t_/C_0_) × 100%, (1)

where C_t_ is the cumulative amount of APC released in the release medium at time t and C_0_ is the initial amount of drug in the APC-SNEDDS. The obtained results were analyzed for drug release kinetics using Microsoft Excel 2010 (version number 14.0.6023.1000).

### 2.11. Evaluation of Antifungal Activity

The NE obtained from aqueous dilution of APC-SNEDDS having an APC concentration of 1.6 mg APC/mL was used to evaluate the antifungal activity of APC-SNEDDS in comparison to a nystatin suspension (1 mg/mL). *C. albicans* ATCC 90028 obtained from the Thailand Network on Culture Collection (Pathum Thani, Thailand) was used in this study. The inoculum of *C. albicans* was cultured in SDB media and incubated at 37 °C for 24–48 h. Before antifungal evaluation, the test sample mixtures were prepared as follows: 500 µL of the test sample (NE of APC-SNEDDS or nystatin suspension) was mixed with 500 µL of *C. albicans* suspension having a concentration of 1 × 10^4^ colony forming unit (CFU)/mL. The prepared samples were tested for killing kinetic and the destructive effects on *C. albicans* cells. A blank sample as a control was prepared in the same manner as the test samples, using SDB instead of APC-SNEDDS.

#### 2.11.1. Killing Kinetics

The test samples and blank were incubated at 37 °C. Samples were withdrawn at the time intervals of 0, 60, 120, 240, 360, and 480 min. Determination of viable *C. albicans* cells was done by spreading 20 μL of the known dilutions of the withdrawn samples on the surface of SDA plates. After that, the plates were incubated at 37 °C for 24–48 h. The colonies of the retained *C. albicans* cells were determined and the results were expressed as Log CFU/mL. Killing kinetic curves of the samples were constructed by plotting Log CFU/mL vs. time.

#### 2.11.2. Effects on Destruction of Candida Cells

The test samples and blank were incubated at 37 °C for 24 h. After that, they were filtered through a nylon membrane (Whatman, Marlborough, MA, USA) and the attached *C. albicans* cells were fixed with 2% *v*/*v* glutaraldehyde in phosphate buffer solution (pH 7.4, 137 mM NaCl, 10 mM Na_2_HPO_4_, 2.68 mM KCl, and 1.84 mM KH_2_PO_4_). The cells were washed to remove glutaraldehyde and other suspended agents with the same buffer solution. Then, the cells were dehydrated in increasing concentrations of ethanol (50, 70, 85, 95, and 100% *v*/*v*), for 10 min each. Chips of the nylon membranes covered with the microbial cells after dehydration were dried using a critical point dryer (K850-813, Quorum Technologies Ltd., Lewes, England). The dried chips were then mounted on an aluminum stub and coated with gold using a sputter coater (JEOL JFC-1100E, Tokyo, Japan) and observed by SEM (JEOL JSM-6610LV, Tokyo, Japan) using at 15 kV accelerating voltage at a magnification of 5000×.

### 2.12. Cytotoxicity to Fibroblast Cells

Investigation of the cytotoxicity to fibroblast cells of the human periodontal ligament was performed using MTT assay following the method of Schmidt et al. [23] with some modifications. The NE obtained from aqueous dilution of APC-SNEDDS with the same APC concentration as used in the antifungal activity study (1.6 mg/mL) was investigated. An NE of SNEDDS without APC, at the same amount used for APC-SNEDD (11.2 mg), was investigated in comparison. The cells were cultured in completed DMEM and incubated at 37 °C in a 5% CO_2_ humidified atmosphere. Then, the cells were seeded into 96-well plates to obtain a density of 10,000 cells/well and further incubated for 24 h at the same conditions. To evaluate the cytotoxicity, the cells were treated with the NE of APC-SNEDDS and SNEDDS. Cytotoxicity was determined at 5, 10, 20, 30, and 60 min. After each timepoint, the supernatant was removed, and the wells were washed with PBS. Then, 100 µL of MTT solution at a concentration of 0.5 mg/mL in PBS was added to the wells, followed by further incubation for 4 h. The MTT solutions were removed and 50 µL of DMSO was added to dissolve the formed formazan crystals. After 15 min, absorbance was measured at 540 nm using a microplate reader (Tecan, Männedorf, Switzerland). The results were calculated and expressed as the percentage of cell viability.

### 2.13. Statistical Analysis

The results are expressed as mean ± S.D. (*n* = 3). Statistical analysis was performed on SPSS software version 17.0 for Windows. Differences between groups were determined by one-way analysis of variance (ANOVA) followed by Tukey’s post-hoc test. A *p*-value ≤ 0.05 was considered as statistically significant.

## 3. Results and Discussion

### 3.1. Solubility of APC and Selection of Excipients

**Figure 2:**
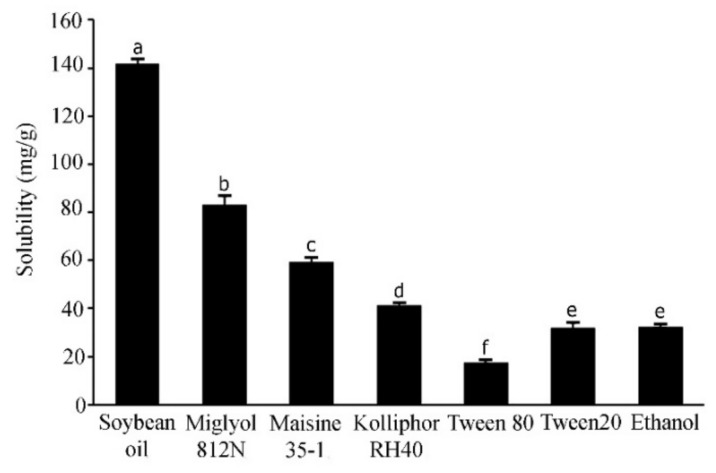
Solubility of APC in various excipients. Different letters indicate significance levels at *p* < 0.05.

The solubility of active compounds in individual excipients is an important prerequisite to prepare the optimized SNEDDS formulations with a high drug loading [24]. In the current study, the solubility of APC in the various excipients was calculated from the linear equation obtained from HPLC analysis (y = 0.134x − 0.5426) of a standard APC solution; (r^2^ = 0.9998). The results are shown in Figure 2. APC was highly soluble in soybean oil, a long-chain triglyceride (141.6 ± 12.30 mg/g), followed by Miglyol 812N, a medium-chain triglyceride (83.0 ± 3.86 mg/g), and Maisine 35-1, a co-surfactant (59.16 ± 2.23 mg/g). The surfactants used in this experiment are non-ionic with a comparatively low toxicity [25]. These surfactants have been reported to promote the formation of stable o/w emulsions with fine droplets due to their hydrophilic–lipophilic balance (HLB) values of approximately 12 or higher [26,27]. In the present study, the surfactant that could provide the highest solubilization capacity for APC is Kolliphor RH40 (41.19 ± 1.10 mg/g), followed by Tween 20 (31.91 ± 2.02 mg/g) and Tween 80 (17.17 ± 0.79 mg/g). These three surfactants have HLB values of 14–16, 16.7 and 15, respectively [28]. Cosolvents in SNEDDS formulations are used to enhance the dispersibility of hydrophilic surfactants in the oil phase, promoting the formation of a homogenous and stable formulation [29]. The present study showed that whilst the solubility of APC in water is only 4.88 ± 0.53 mg/mL, it is about 8-fold higher (32.06 ± 0.54 mg/g) in absolute ethanol. Alcohol is generally considered to be a good cosolvent, with a low viscosity, to reduce emulsification time to less than 60 s [30]. Therefore, absolute ethanol was selected in the present study to be used as a cosolvent for the SNEDDS systems. Previous studies have shown the successful use of mixed ingredients comprising oils, surfactants, co-surfactant, and cosolvent in the development of SNEDDS formulations. For example, SNEDDS comprising soybean oil, Maisine 35-1, Kolliphor RH40, and ethanol for fenofibrate [19], SNEDDS composed of soybean oil or rapeseed oil, Kolliphor RH40, Maisine35-1, and ethanol for halofantrine [31], and SNEDDS composed of Miglyol 812N, Kolliphor RH40, Capmul MCM C10, and ethanol to increase the transepithelial permeability of insulin [32]. In the present study, the solubility tests suggest that high solubility of APC can be obtained from soybean oil, Miglyol 812N, Maisine 35-1, Kolliphor RH40 and absolute ethanol. Therefore, these excipients were selected to develop SNEDDS formulations with a high loading capacity for APC.

### 3.2. Preparation of SNEDDS Formulations

The selected excipients from the solubility study were used to generate an experimental matrix for designing optimized SNEDDS. As shown in Table 2, 14 SNEDDS formulations were generated from the D-optimal design. These formulations can be categorized into two groups: a group of long-chain triglyceride systems (with soybean oil, F1–F7) and a group of medium-chain triglyceride systems (with Miglyol 812N, F8–F14). After mixing all excipients, each of these anhydrous formulations was a clear and homogenous mixture without phase separation.

### 3.3. Self-Emulsification Efficiency and Particle Characteristics

A good SNEDDS formulation should present self-emulsification after contact with water or aqueous systems without any sign of emulsion instability such as coalescence, creaming, flocculation, and breaking [33]. In the present study, after dispersing the formulated SNEDDS formulations in Milli-Q water at ambient temperature, it was found that the obtained mixtures can be categorized into four groups of grade A, B, C, and D. Grade A formulations are the most effective to form the desired NEs with a clear or bluish appearance [20]: two long-chain triglyceride formulations, F4 and F7, and three short-chain triglyceride formulations, F9, F11, and F14.

Particle characterization was performed after diluting all formulated SNEDDS in water to obtain a 1:100 dilutions. The particle size and size distribution of the oil droplets of each dilution were determined using PCS [34]. The results presented in Table 3, as expected, suggest that the particle size of the obtained formulations is related to the grading of the systems. The formulations in grade A, F4, F7, F9, F11, and F14 demonstrate a small particle size of no more than 100 nm. These formulations also show a narrow PdI value of not more than 0.22, indicating a comparatively high uniformity of the particle size in the systems [35]. From these results, it can be concluded that the best self-formed transparent o/w NEs can be obtained from SNEDDS formulations F4, F7, F9, F11, and F14 with uniform droplet distributions of less than 100 nm.

### 3.4. Solubility of APC in SNEDDS Formulations

**Figure 3:**
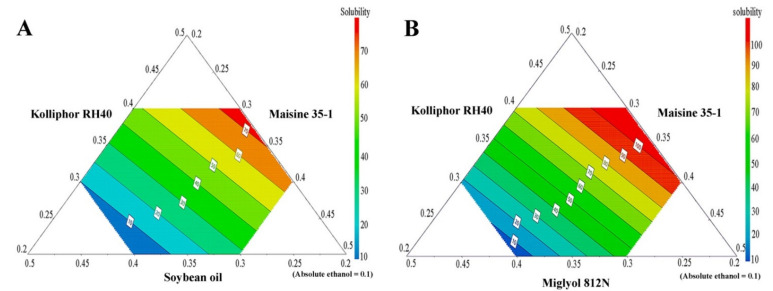
The prediction contour plots of APC solubility in SNEDDS formulations; long-chain triglyceride SNEDDS system (**A**) and medium-chain triglyceride SNEDDS system (**B**).

APC solubility in the various SNEDDS is shown in Table 4, and, together with the compositions of the SNEDDS, was used to generate prediction contour plots. The results, shown in Figure 3, demonstrate large variations in APC-loading capacities for different formulations, depending on the type and concentration of the excipients used. From these results, it can be concluded that SNEDDS formulations containing a medium-chain triglyceride (Miglyol 812N) have a higher ability to solubilize APC than the systems containing a long-chain triglyceride (soybean oil). This result is in agreement with previous work in which the medium-chain triglyceride (Capmul MCM) showed a higher ability to enhance the solubility of the drug carvedilol than the long-chain triglycerides (Labrafil M 2125 and Labrafil M 2130) [36].

The results of the solubility study (and the resulting contour plots), however, also indicate that formulations containing a high amount of Kolliphor RH40 have a significantly higher APC-loading capacity. Therefore, it can be concluded that Kolliphor RH40 plays a major role in the ability of SNEDDS formulations to enhance APC solubility and loading capacity. These results are supported by previous studies in which Kolliphor RH40 was used as a potential surfactant in SNEDDS system to enhance the solubility of hydrophobic drugs such as irbesartan [37], atorvastatin calcium [38], and candesartan cilexetil [39].

### 3.5. Preparation and Characterization of APC-SNEDDS

The SNEDDS formulation F11 was selected for further study because APC showed the highest solubility in this formulation and because it is furthermore categorized as grade A. An NE with a clear or bluish appearance could be formed within 1 min after dispersion in water and the obtained NE showed the smallest particle size and narrowest size distribution of the internal phase. The solubility of APC in F11 (141.48 ± 15.64 mg/g) corresponds to a weight ratio of APC to SNEDDS of approximately 1:7. This ratio, therefore, was used to prepare APC-SNEDDS to obtain clear NEs at three APC concentrations: 0.4, 0.8, and 1.6 mg/mL. Based on our previous study [8], these concentrations correspond to 1-fold, 2-fold, and 4-fold of the minimum fungicidal concentration against *C. albicans*. It is noted that the apparent solubility of APC in water for this NE is 141.48 ± 15.64 mg/mL, compared to a solubility of APC in water of 4.88 ± 0.53 mg/mL. This result obviously indicates that SNEDDS formulation F11 can effectively enhance the water solubility of APC.

The particle size, PdI, and zeta potential of the obtained NEs having APC concentrations of 0.4, 0.8, and 1.6 mg/mL are shown in Table 5. It is found that the particle size of the NEs from APC-SNEDDS was in the range of 33–35 nm, which is slightly larger than for the blank SNEDDS (28.28 ± 0.36 nm), likely due to the entrapped APC inside the droplets. The particle size and size distribution of the NEs for the three APC concentrations showed no significant difference (*p* > 0.05) since the same ratio of APC:SNEDDS was used for each APC concentration and all APC can dissolve freely in the SNEDDS at the used ratio of 1:7. The PdI of the three formulations is in the range of 0.14–0.15 and was not significantly different from the blank (0.11 ± 0.02). The low PdI values indicate that the NE systems possess good stability and size uniformity [40].

The NEs obtained from the selected SNEDDS formulation had a negative zeta potential (−9.73 ± 1.62 mV), probably due to the ionization of free fatty acids of Kolliphor RH40 [39,41]. Moreover, it is noticed that the zeta potential values of the droplets from APC-SNEDDS were between −16.20 ± 1.31 mV and −20.37 ± 2.17 mV, significantly higher than that obtained from blank SNEDDS. The high negative zeta potential of the droplets from APC-SNEDDS is probably due to the ionization of phenolic OH– group of APC in the system [42,43].

### 3.6. Morphology Study

**Figure 4:**
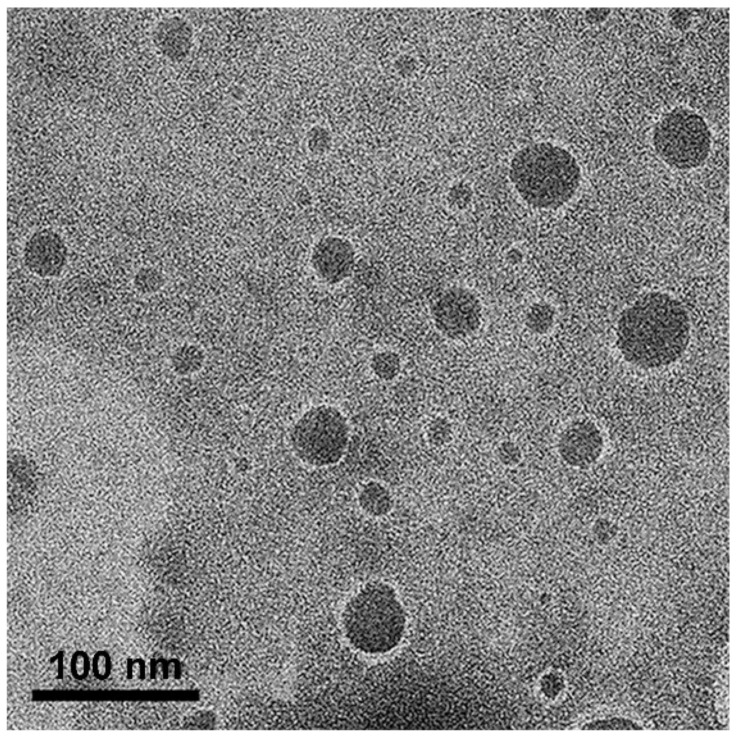
TEM images of selected APC-SNEDDS on phase behavior of oil in water systems (50,000×).

The morphology of the oil droplets in the NEs obtained from the APC-SNEDDS was investigated by TEM. The TEM images, as shown in Figure 4, demonstrated the particles as spherical dark globules with a smooth surface and good dispersion. The particle size observed from TEM is closed to that obtained by PCS. However, some big particles are found, which may be due to the dehydration process of sample preparation for electron microscopy, and led to droplet aggregation [44].

### 3.7. In Vitro Release

**Figure 5:**
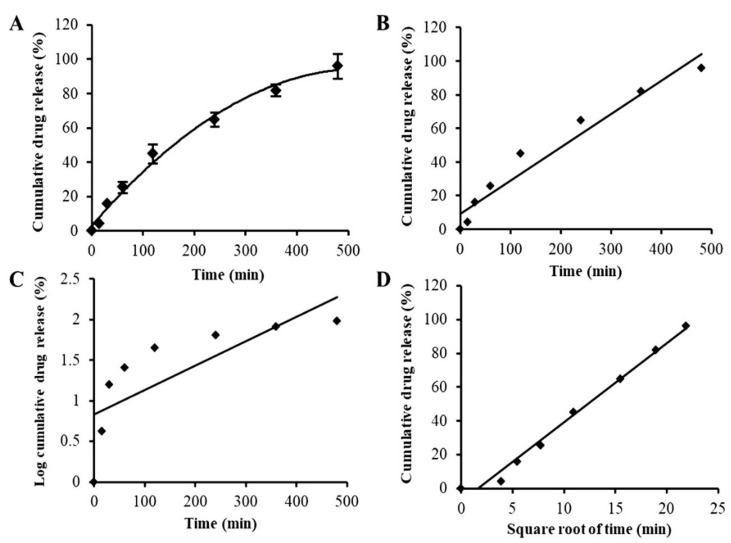
In vitro drug release profile of APC-SNEDDS (**A**) and drug release kinetics plots using various kinetic models: zero-order kinetics (r^2^ = 0.9513) (**B**), first-order kinetics (r^2^ = 0.6008) (**C**), and Higuchi model (r^2^ = 0.9875) (**D**).

Artificial saliva was used as medium in the in vitro release study. The release study was conducted under sink conditions. The drug release profile of APC-SNEDDS, as shown in Figure 5A, demonstrated a short initial burst release of 5.89 ± 0.32% after the first 30 min of the experiment and subsequent sustained released. The release of APC at the end of the experiment (480 min) was found to be 95.98 ± 7.15%. This release behavior is similar to a previous report [45] and can be considered as a sustained release.

Drug release kinetics can be governed by one or more mechanisms depending on the composition of the matrix, the preparation method, and the dissolution media of drug release. Many mathematical models are used to describe drug release kinetics and to predict the release behavior of the drug [46]. In the present study, the release mechanism of APC from SNEDDS was evaluated by three mathematical models: the zero order, first order, and Higuchi models. These mathematical models can suggest whether the drug release is dependent on the drug concentration (first-order kinetics) or the initial drug concentration at a constant rate (zero-order kinetics). The Higuchi model represents the release of drugs from an insoluble matrix based on Fickian diffusion, which is commonly found in nanoparticles [47]. Suitable mathematical models can be chosen from the value of the correlation coefficient (r^2^) obtained from the linear regression analysis according to each model as shown in Figure 5B–D, respectively. The results indicated that the release behavior of APC from APC-SNEDDS was best fitted to the Higuchi model, suggesting that the drug release is likely to be according to a Fickian diffusion mechanism. However, simply basing the release mechanism on the different r^2^ values of the models might be insufficient, and further studies on the exact release mechanism are warranted. 

### 3.8. Antifungal Activity

#### 3.8.1. Killing Kinetics

**Figure 6:**
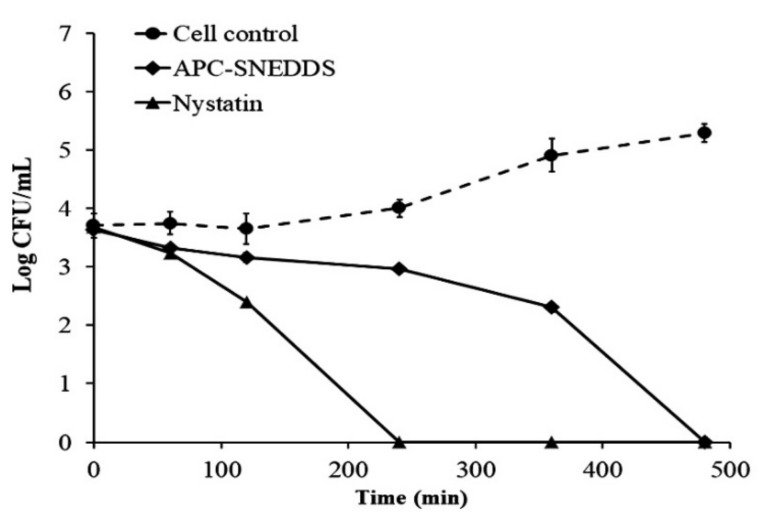
Killing kinetics curves of APC-SNEDDS and nystatin against *C. albicans* in comparison with control cells (untreated).

The killing kinetic profiles against *C. albicans* are shown in Figure 6 and demonstrate that the NE obtained from the APC-SNEDDS possessed effective antifungal potential against *C. albicans*. The NE of the selected APC-SNEDDS significantly reduced the number of viable cells of *C. albicans* (*p* < 0.05) when compared to the cell control. The reduction in cell viability is more than 90% in 240 min, which is equivalent to more than 1 Log CFU/mL. The killing kinetic of the NE obtained from the APC-SNEDDS showed sustained activity against *C. albicans* and showed complete killing within 480 min. The killing kinetic of nystatin showed complete killing within 240 min. However, our previous study found that the antifungal activity of APC was concentration-dependent [8]. Thus, the activity of the NE obtained from the APC-SNEDDS can be increased to a higher potency by increasing APC-SNEDDS concentration in water.

#### 3.8.2. Effect on the Destruction of Candida Cells

**Figure 7:**
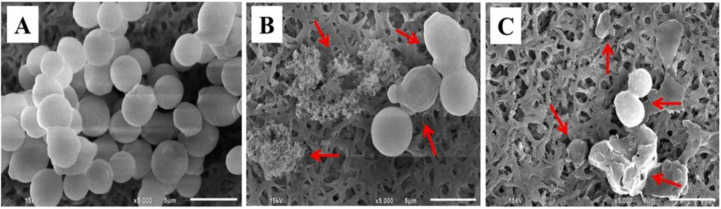
SEM micrographs of *C. albicans* cells. Untreated cells (**A**), cells treated with APC-SNEDDS (**B**), and cells treated with nystatin (**C**) (5000×). Arrows indicate the irreversible morphological destruction of the treated *C. albicans* cells.

*C. albicans* exhibits widely different morphological phenotypes which depend on bud to hypha transition and phenotypic switching [48]. Morphological alteration of *C. albicans*, observed by SEM, is presented in Figure 7. The untreated cells (control cells) had a smooth surface with a spherical shape (Figure 7A). In contrast, *Candida* cells treated with the NE obtained from the APC-SNEDDS exhibited an altered surface roughness, cell swelling, disruption of phenotypic switching, and cell damage (Figure 7B). The morphology of *C. albicans* cells after exposure to nystatin is different (Figure 7C), as the cells exhibited no swelling before cell disruption. Based on these morphological differences, the mechanism of action of APC and nystatin appears to be different. The antifungal mechanism of nystatin is due to the formation of pores at the cell membrane by binding to ergosterol, leading to K+ leakage and contribution of cell death [49,50]. However, the antifungal mechanism of APC is the generating superoxide or free radicals from the phenolic group to damage DNA of the fungal cells [51].

### 3.9. Cytotoxicity to Fibroblast Cells

**Figure 8:**
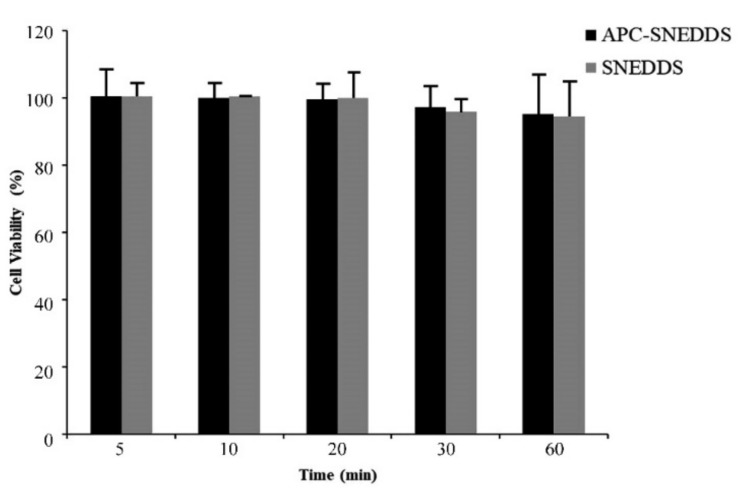
Viability of fibroblast cells from the human periodontal ligament after exposure to APC SNEDDS (black columns) and the blank SNEDDS (grey columns).

A cytotoxicity test of APC-SNEDDS to fibroblast cells extracted from human periodontal ligament was performed using MTT assay. MTT is a yellow water-soluble tetrazolium dye but, after reacting with mitochondrial succinate dehydrogenases that are found in mitochondria of living cells, formazan crystals are formed [52]. Cell viability is determined by the quantitative analysis of the formazan crystals [53]. The results of this study are shown in Figure 8. It was found that NE of APC-SNEDDS containing 1.6 mg/mL APC showed no significant loss of fibroblast cell viability after 5-, 10-, 20-, 30-, and 60-min exposure. Generally, a test sample showing cell viability above 80% is regarded as safe or non-toxic to the cells [54]. Our results indicate that APC-SNEDDS with APC at an effective concentration are non-toxic to fibroblast cells. For this reason, it can be concluded that APC-SNEEDS are a promising potential candidate for oral applications.

## 4. Conclusions

In the present study, 4-allylpyrocatechol (APC) loaded SNEDDS (APC-SNEDDS) were successfully prepared, with significantly enhanced apparent solubility. The optimized SNEDDS formulation is composed of 20% Miglyol 812N, 40% Kolliphor RH40, 30% Maisine 35-1 and 10% absolute ethanol. This formulation provides the highest loading capacity of APC of the tested formulations. A clear solution with no signs of precipitation or separation formed self-nanoemulsification in water. The release of APC-SNEDDS followed the Higuchi model. The developed APC-SNEDDS formulation is nontoxic to normal fibroblast cells and effectively inhibits *C. albicans*, the main cause of oral candidiasis.

## Figures and Tables

**Table 1:** Lipid classification systems, reprinted with permission from [13], Taylor & Francis, 2017.

Types	Excipients in Formulation (% *w*/*w*)				Characteristics
	Oils: Glycerides/ Mixed Glycerides	Lipophilic Surfactants (HLB < 12)	Hydrophilic Surfactants (HLB > 12)	Co-Surfactants	
I	100	-	-	-	Pure oils, limited or no dispersion
II	40–80	20–60	-	-	Moderate dispersion needed to form an emulsion
IIIA	40–80	20–40	-	0–40	Rapid dispersion to form micro- or nano-emulsion
IIIB	<20	-	20–50	20–50	Rapid dispersion to form micro- or nano-emulsion
IV	-	0–20	20–80	0–80	Oil free, rapid dispersion results in micellar solution

Abbreviations: HLB, hydrophilic-lipophilic balance.

**Table 2:** Composition of self-nanoemulsifying drug delivery system (SNEDDS) formulations that were operated by D-optimal design using Umetric modeling and design (MODDE) software.

Formulations	Excipients (% *w*/*w*)				
	Soybean Oil	Miglyol 812N	Kolliphor RH40	Maisine 35-1	Absolute Ethanol
F1	30	-	20	40	10
F2	30	-	40	20	10
F3	20	-	30	40	10
F4	20	-	40	30	10
F5	40	-	20	30	10
F6	40	-	30	20	10
F7	30	-	30	30	10
F8	-	30	20	40	10
F9	-	30	40	20	10
F10	-	20	30	40	10
F11	-	20	40	30	10
F12	-	40	20	30	10
F13	-	40	30	20	10
F14	-	30	30	30	10

**Table 3:** The effects of different concentrations of excipients in SNEDDS formulations on self-emulsification efficiency, particle size, and size distribution (PdI) of the obtained nanoemulsions.

Formulations *	Self-Emulsification Efficiency	Particle Size ** (nm)	PdI **
F1	C	317.24 ± 1.34 g,h	0.27 ± 0.08 d,e
F2	D	178.40 ± 0.42 f	0.20 ± 0.03 c,d
F3	B	158.50 ± 1.57 e,f	0.24 ± 0.13 c,d
F4	A	40.42 ± 0.76 a	0.12 ± 0.02 a,b
F5	D	255.00 ± 0.99 g	0.59 ± 0.00 f
F6	C	347.40 ± 4.34 h	0.18 ± 0.09 a,b,c,d
F7	A	65.82 ± 0.22 a,b	0.22 ± 0.01 b
F8	B	490.80 ± 21.43 i	0.09 ± 0.05 a
F9	A	52.57 ± 1.35 a	0.14 ± 0.01 a,b
F10	B	136.07 ± 7.84 e	0.38 ± 0.06 f
F11	A	28.28 ± 0.36 a	0.11 ± 0.02 a
F12	C	329.90 ± 9.28 h	0.14 ± 0.03 a,b,c
F13	B	115.53 ± 1.62 c	0.37 ± 0.01 e,f
F14	A	98.98 ± 1.62 c	0.15 ± 0.00 a,b,c

* Visually all systems were homogenous and did not show any phase separation. ** Lowercase letters indicate significant differences (*p* < 0.05) for the particle size and PdI analysis of the different formulations.

**Table 4:** Solubility of APC in SNEDDS formulations.

Formulations	Solubility of APC in SNEDDS (mg/g) *
F1	38.73 ± 2.79 f
F2	65.45 ± 1.57 d
F3	91.82 ± 2.06 b
F4	77.97 ± 7.01 b,c
F5	8.75 ± 1.34 g
F6	10.45 ± 0.95 g
F7	12.36 ± 1.61 g
F8	48.01 ± 8.78 e
F9	72.55 ± 0.10 c,d
F10	91.10 ± 9.01 b
F11	141.48 ± 15.64 a
F12	45.87 ± 6.31 e,f
F13	19.77 ± 1.13 g
F14	18.19 ± 2.39 g

* Lowercase letters indicate significant difference (*p* < 0.05) for solubility values of the different formulations.

**Table 5:** Particle size analysis of APC-SNEDDS that contained various concentrations of APC.

APC Concentration (mg)	Particle Size * (nm)	PdI *	Zeta Potential * (mV)
0.4	35.22 ± 0.43 a	0.15 ± 0.04 a	–17.13 ± 1.80 b
0.8	34.59 ± 0.30 a	0.21 ± 0.01 b	–16.20 ± 1.31 b
1.6	33.00 ± 0.30 a	0.14 ± 0.01 a	–20.37 ± 2.17 a

* Lowercase letters indicate significant difference (*p* < 0.05) for solubility values of the different formulations.

## Data Availability

All data are available upon request.
